# Comparison of different hot air segmented drying techniques for peanut pods: changes in drying characteristics, microstructure, moisture migration, nutritional quality and flavor quality

**DOI:** 10.1016/j.fochx.2026.104533

**Published:** 2026-09-29

**Authors:** Pengxiao Chen, Siyuan Zhao, Wenxue Zhu, Tingting Zhang, Kai Zhang, Yanjia Xing, Can Hua, Debang Zhang

**Affiliations:** a School of Food and Strategic Reserves, Henan University of Technology, Zhengzhou 450001, China; b School of Electromechanical Engineering, Henan University of Technology, Zhengzhou 450001, China; c Zhengzhou Wangu Machinery Co.,Ltd.,Zhengzhou 450041, China

**Keywords:** Peanut pods, Hot air drying, Segmented drying, Drying characteristics, Flavor

## Abstract

This study investigated the impact of various hot air segmented drying (HASD) methods on peanut pods. The drying characteristics, microstructure, moisture migration, nutritional and flavor qualities of peanut pods were comparatively evaluated using hot air-heat pump segmented drying (HAD-HPD), hot air-infrared segmented drying (HAD-IRD), hot air-radio frequency segmented drying (HAD-RFD), hot air drying (HAD) and natural drying (ND). The results indicated that HASD exhibited a transition point in the drying mechanism where the drying rate changed markedly with the shift in drying mode. After the transition point, HAD-IRD and HAD-RFD promoted synchronous outward migration of moisture from both the inner and outer layers. For nutrition, excluding the ND group, HAD-IRD had the lowest acid value (0.94 ± 0.04 mg/g), the lowest peroxide value (0.062 ± 0.001 g/100 g) and the highest amino acid content (26.37 ± 0.20%). HAD-RFD kernels exhibited the highest concentrations of volatile organic compounds (VOCs). Principal component analysis (PCA) suggested that HASD was superior to HAD, and HAD-IRD performed best among them.

## Highlights

• HASD has a unique drying mechanism transition point, improving late-stage drying rate and exceeding hot-air and natural drying for peanut pods.
• HAD-IRD achieves superior nutritional quality and is recommended as the optional drying method for high-quality peanut pods.
• HAD-RFD accelerates internal and external moisture migration but induces tissue structural damage and undesirable off-flavors.

## 1 Introduction

As a typical pericarp-kernel bilayer agricultural product, peanut pods possess a dense shell that acts as a significant barrier to heat and mass transfer, resulting in drying characteristics that differ greatly from those of single-grain crops. Freshly harvested peanut pods contain approximately 45% moisture, rendering them susceptible to heat generation, mould growth, and aflatoxin contamination due to vigorous respiration (Mdindikasi et al., 2024). On a global scale, approximately 10% of the total peanut pod yield is lost annually due to delayed drying, representing a financial loss exceeding 10 billion US dollars (Prajapati & Sheorey, 2022). Drying can facilitate timely moisture reduction, thereby inhibiting the growth of microbes and extending storage life. Furthermore, drying is a prerequisite for subsequent processing and transportation (Zhang, Wang, et al., 2025). Drying plays a crucial role in the peanut industry by reducing mould contamination and minimizing post-harvest losses. This process is instrumental in safeguarding food security, thereby laying a solid foundation for the stable and sustainable development of the peanut industrial chain.

At present, the most common drying methods for peanut pods are natural drying (ND) and hot air drying (HAD). Furthermore, a range of drying techniques have been developed and applied, including heat pump drying (HPD), infrared drying (IRD), radio frequency drying (RFD), and microwave drying (MWD). ND is limited by meteorological conditions, resulting in protracted drying times, high labor intensity and low efficiency. Hot air drying offers simple equipment configuration and easy operation. However, the migration of moisture within the material during drying proceeds in the opposite direction to that of heat transfer (Yan et al., 2021), resulting in inefficient drying and loss of nutritional components (Qu et al., 2020).Heat pump drying is gentle and controllable, thereby preventing high-temperature damage to heat-sensitive materials. However, the equipment is complex and requires substantial initial investment (Zhu et al., 2025).Infrared drying offers efficient heat transfer, rapid temperature rise, and low energy consumption, though the appearance and quality of the dried material are generally moderate (An et al., 2025). Radio frequency drying offers strong penetration, efficient and uniform heating, and preserves both nutritional value and appearance. However, this technique may induce edge effects, which can result in scorching and undesirable flavors (Mao & Wang, 2023). Microwave drying (MWD) offers high efficiency and a strong ability to retain nutrients. However, it readily creates internal and external temperature gradients, leading to surface overheating and even cracking. In summary, single-segmented drying techniques are unable to balance drying efficiency, quality, and costs. Current studies on peanut drying mainly focus on various combined drying techniques, such as radio frequency-vacuum combined drying(Lei et al., 2025), hot air-microwave combined drying (An et al., 2024), and hot air-radio frequency combined drying (Chen, Li, Zhu, et al., 2025). Systematic research targeting peanut pod segmented drying is still limited. Studies on segmented drying techniques for agricultural products have shown that hot air-infrared segmented drying facilitated the conversion of bound water and weakly bound water within peanut pods, while also removing free water, thereby reducing drying duration. Radio frequency-hot air segmented drying could reduce the drying time of goji berries while preserving nutritional components such as total sugar, total phenolic content, flavonoid content and vitamin C more effectively (Xu, Pei, et al., 2022). Heat pump-infrared segmented drying reduced the loss of total amino acids and essential amino acids in peanut pods while preserving their superior internal microstructure (Chen, Wang, Zhu, et al., 2025). Microwave-hot air segmented drying enhanced the anthocyanin content and antioxidant capacity of purple cabbage, increasing its rehydration rate to 87.59% (Liu et al., 2021). Hot air radio frequency segmented drying enhanced the retention of soluble sugars, vitamin C, and color in mangoes, yielding a quality comparable to vacuum drying while achieving a significant reduction in costs. Hot air-vacuum segmented drying preserved the phospholipid and astaxanthin contents of krill, while concurrently exhibiting effective inhibition of astaxanthin isomerisation and oxidation of unsaturated fatty acids (Zheng et al., 2024). Despite these findings, few studies have systematically compared multiple hot air-based segmented drying approaches for peanut pods. Considering the physical characteristics of peanut pods, drying performance, nutrient protection and industrial applicability, we selected HPD, IRD and RFD to combine with HAD. Other drying techniques such as microwave drying and vacuum drying were excluded due to their limitations in operational stability and production cost for peanut processing.

The present study aims to investigate the merits and drawbacks of HASD for peanut pods. To this end, the drying characteristics, microstructure, moisture migration, nutritional quality, and flavor characteristics of peanut pods subjected to different drying treatments, including ND, HAD, HAD-HPD, HAD-IRD, and HAD-RFD, were examined. A comprehensive scoring method was employed to evaluate the different drying techniques to identify the optimal HASD for peanut pods. Unlike previous studies that predominantly analyzed individual quality indicators separately, the comprehensive scoring method integrates multiple quality parameters into a unified evaluation system via PCA, enabling an intuitive overall comparison of different drying treatments and thereby highlighting the innovation of this work. This study provides novel insights for the industrial application of peanut pod drying.

## 2 Materials and methods

### 2.1 Materials

Freshly harvested peanut pods (cultivar “Huayu 25”) were purchased in October 2024 from local farmers in Shangqiu City, Henan Province, China. Before the experiment, the samples were selected to ensure intact shells and uniform size. They were subsequently thoroughly washed and stored in a refrigerated storage chamber at 4 °C. The initial moisture content of the fresh peanut pods on a dry basis (d.b.) was 0.9428 g/g.

### 2.2 Drying experimental design

#### 2.2.1 Experimental design

The thin-layer drying method was adopted in this experiment, where peanut pods were placed in a single layer on a mesh tray for drying with no overlap. During the experiment, the average ambient temperature was 28 °C and the average humidity was 55%. For HAD, the direction of the hot air flow was perpendicular to the peanut pods. For IRD, the infrared radiation distance was 12 cm. For RFD, the radio frequency radiation distance was 12 cm. As shown in Table 1, formal single-factor drying trials were conducted on peanut pods using ND, HAD, HAD-HPD, HAD-IRD, and HAD-RFD methods to investigate their drying characteristics with variable gradients determined based on preliminary trials. As shown in Table 2, the optimal drying parameters were determined through preliminary response surface methodology (RSM) experiments. Based on these pre-experimental optimization results, the optimal drying scheme was applied for formal drying treatments and subsequent quality evaluation.

**Table 1:** Single-factor drying test.

Drying method	Drying parameters	Equipment
ND	Lay out in a sunny, well-ventilated spot, turning regularly, and bring indoors at night.	/
HAD	Fixed air velocity at 1.2 m/s, Set hot air temperature between 36 °C to 48 °C (gradient: 3 °C). Fixed hot air temperature at 42 °C, Set hot air velocity between 0.3 m/s to 1.5 m/s (gradient: 0.3 m/s).	Hot air dryer, JK-LB1700, Changchun Shenyang Mechanical and Electrical Co., Ltd., Changchun, China
HAD-HPD	Fixed conversion moisture content at 35%, heat pump temperature at 55 °C, with hot air temperature set between 35 °C and 55 °C (5 °C gradient). Fixed hot air temperature at 45 °C, heat pump temperature at 55 °C, with conversion moisture content set between 25% and 45% (5% gradient). Fixed hot air temperature at 45 °C, conversion moisture content at 35%, with heat pump temperature set between 45 °C and 60 °C (5 °C gradient).	Air-source heat pump dryer, AGHD-1SELC, Aoyimei Energy Saving Technology Co., Ltd., Shanghai, China
HAD-IRD	Fixed conversion moisture content at 30%, infrared temperature at 50 °C, with hot air temperature set between 35 °C and 55 °C (5 °C gradient). Fixed hot air temperature at 45 °C, infrared temperature at 50 °C, with conversion moisture content set between 20% and 40% (5% gradient). Fixed hot air temperature at 45 °C, conversion moisture content at 30%, set infrared temperature from 40 °C to 60 °C (gradient of 5 °C).	Infrared oven, ZKT-210EF, Bafunuo Mechanical Equipment Co., Ltd.,Nanjing, China
HAD-RFD	Fixed conversion moisture content at 35%, RF power at 1.5 kW, with hot air temperature set between 35 °C and 55 °C (5 °C gradient). Fixed hot air temperature at 45 °C, RF power at 1.5 kW, with conversion moisture content set between 25% and 45% (5% gradient). Fixed hot air temperature at 45 °C, conversion moisture content at 35%, with RF power set between 0.5 kW and 2.5 kW (0.5 kW gradient).	Radio frequency dryer, HGJL-10RFS, Hefei Harbin Institute of Technology Jinlang Equipment Technology Co., Ltd., Hefei, China

**Table 2:** Optimal drying parameters.

Drying method	Drying parameters	Drying Time
HAD	Hot air temperature: 42 °C	HAD for 28 h
HAD-HPD	Hot air temperature: 45 °C, Conversion moisture content: 34%, Infrared temperature: 56 °C	HAD for 6 h, HPD for 15 h
HAD-IRD	Hot air temperature: 46 °C, Conversion moisture content: 30%, Infrared temperature: 51 °C	HAD for 8 h, HPD for 12 h
HAD-RFD	Hot air temperature: 50 °C, Conversion moisture content: 40%, Radio frequency power: 1.6 kW	HAD for 5 h, HPD for 11 h

#### 2.2.2 Drying procedure

For HASD, 200 g of peanut pods were placed in the hot air dryer initially. Upon reaching the predefined moisture content transition point, the samples were transferred to the corresponding subsequent equipment (air-source heat pump dryer, infrared oven and radio frequency dryer) for continued drying until the dry basis moisture content reached 10% (d.b.). During drying, the samples' weights were recorded every hour; when approaching the moisture content transition point, the recording interval was reduced to 30 min. For HAD, no transfer between drying equipment was required. For ND, the samples' weights were recorded every 24 h, and the recording interval was switched to every 2 h during the final drying stage.

### 2.3 Measurement of drying characteristics

Moisture content on a dry basis (M_t_) and drying rate (DR), were calculated using eqs. (1), (2) respectively，

(1) $$M_{t}=\frac{W_{t}-m}{m}$$

(2) $$\begin{array}{c} \mathrm{DR}=\frac{M_{t}-M_{t+△t}}{△t} \end{array}$$

where M_t_, the dry basis moisture content (g/g); W_t_, the mass dried to any t moment (g); m, the mass of dry matter (g); DR, the drying rate (g/(g·h)); and M_t+Δt_, the dry basis moisture content at the moment t + Δt (g/g).

### 2.4 Determination of microstructure

Peanut kernels were sliced into uniform thin sections of 60 μm thickness, immersed in petroleum ether for degreasing for 24 h, subsequently freeze-dried for 48 h until fully dehydrated. The sections were flattened and fixed on the sample stage with graphite double-sided adhesive tape, gold-sputtered (sputtering current: 10 mA, duration: 60 s), and transferred to a scanning electron microscope (S-4800, Hitachi Ltd., Japan). The magnification was set to 300× to observe and collect images.

### 2.5 Determination of low-field nuclear magnetic resonance

Peanut kernels were placed in dedicated sample tubes and transferred to a low-field nuclear magnetic resonance imaging system (Variable Temperature NMR Food and Agriculture Imaging Analyzer, MicroMR-CL-I, Niumai Electronic Tech, Shanghai, China) to acquire T₂ inversion spectra and nuclear magnetic resonance imaging maps.

### 2.6 Determination of nutrition characteristics

The nutritional characteristics were determined referring to relevant Chinese national standards with slight modifications, including GB 5009.168–2016 for fatty acids, GB 5009.229–2016 for acid value, GB 5009.227–2016 for peroxide value, GB 5009.5–2016 for protein, GB 5009.124–2016 for amino acids, and GB 5009.6–2016 for fat.

#### 2.6.1 Determination of crude fat

The shelled peanuts were crushed and 2.5 g were placed in a filter paper cylinder. Use the Soxhlet extraction method with petroleum ether as the solvent, conducting reflux extraction at 60 °C for 8 h at a constant velocity. The residue was dried to constant weight and accurately weighed prior to the calculation of crude fat content.

#### 2.6.2 Oil extraction

The shelled peanuts were crushed and 30 g were placed in a stoppered conical flask. 200 mL of petroleum ether were added and the mixture was extracted at 40 °C for 12 h. The mixture was subsequently filtered and the filtrate transferred to a rotary evaporator under reduced pressure. The petroleum ether was subsequently distilled off in order to recover the peanut oil.

#### 2.6.3 Determination of fatty acid composition

Add 0.2 mL of the oil sample to a 50 mL round-bottomed flask. Six milliliters of a 0.5 mol/L sodium hydroxide and methanol solution were subsequently added. Fit a reflux apparatus and slowly heat until boiling. Seven milliliters of 15% boron trifluoride in methanol were subsequently added. After refluxing for 1 min, add 3 mL of n-hexane and continue refluxing for one minute. Once cooled, remove the flask and add a saturated sodium chloride solution. Leave it to stand until the layers separate. Transfer the clear upper layer to a test tube, add anhydrous sodium sulphate and dry. Pipette the dried supernatant into a sample vial for gas chromatography analysis to determine the fatty acid composition.

#### 2.6.4 Determination of acid value

Weigh out 2 g of peanut oil and place it in a conical flask. Add 50 mL of a neutralized solvent mixture of diethyl ether and ethanol (volume ratio 1:1, *v*/v) and shake well to dissolve. Subsequently, two to three drops of phenolphthalein indicator were added, and the solution was titrated with a 0.1 mol/L potassium hydroxide standard solution until a persistent red coloration was observed for 30 s. Record the volume of base consumed and calculate the acid value. A blank titration was performed. A blank titration was performed simultaneously to eliminate solvent interference.

#### 2.6.5 Determination of peroxide value

Weigh 2 g of peanut oil and transfer it to a 250 mL iodometric flask. Add 30 mL of a chloroform and glacial acetic acid solution (volume ratio 2:3, *v*/v), shaking gently until dissolved. Immediately add 1.00 mL of saturated potassium iodide solution, seal the flask tightly, then shake it vigorously for 30 s. Leave to stand undisturbed in the dark for 3 min. Then remove it, add 100 mL of water and shake thoroughly. Titrate promptly with 0.01 mol/L sodium thiosulfate solution until a pale yellow color develops. Add 1 mL of starch indicator and continue the titration until the blue color disappears. Record the volume of titrant used and calculate the peroxide value. A blank titration was performed simultaneously to eliminate solvent interference.

#### 2.6.6 Determination of crude protein

Weigh 2 g of peanut kernels and place them in a digestion tube. Add 0.4 g of copper sulphate, followed by 6 g of potassium sulphate, and subsequently 20 mL of sulphuric acid, in that order. Place the tube in a digestion furnace. Heat to 420 °C and maintain for one hour. Remove once the solution turns green and transparent, then leave to cool. Add 50 mL of water. Use a Kjeldahl nitrogen analyzer to automatically perform liquid addition, distillation, titration and data recording.

#### 2.6.7 Determination of amino acid composition

Weigh 35 mg of defatted peanut powder into a hydrolysis tube. Add 10 mL of 6 mol/L hydrochloric acid and three drops of phenolphthalein. Pass nitrogen through the mixture, then seal the tube. Hydrolyze at 110 °C for 22 h. Once cooled, filter the contents into a 50 mL volumetric flask. Transfer 1 mL of the filtrate to a 50 mL centrifuge tube and evaporate to dryness at 45 °C using a rotary evaporator. Redissolve the residue in 1 mL of ultrapure water, and subsequently dry it by vacuum drying to a constant weight. Dissolve it in 1 mL of sodium citrate buffer, sonicate it for 1–2 min, filter it through a 0.22 μm organic-reactive membrane and transfer it to a sample vial for Automatic Amino Acid Analyzer analysis.

### 2.7 Determination of flavor characteristics

For HS-GC-IMS detection, after crushing, 2.0 g of peanut sample was weighed and placed into a 20 mL headspace vial, followed by the addition of 20 μL 100 mg/L 2-octanol solution as the internal standard; the vial was incubated at 60 °C for 15 min before sample injection, and each biological replicate was determined in triplicate (instrumental replicates). The unified headspace injection parameters were set as follows: incubation temperature of 60 °C, incubation time of 15 min, injection volume of 1000 μL, splitless injection mode, incubation stirring speed of 500 r/min, and syringe temperature of 85 °C. For gas chromatography conditions, a FS-SE-54 capillary column (30 m × 0.25 mm × 0.25 μm) was used for separation. The initial column temperature was maintained at 60 °C, and high-purity nitrogen (purity ≥99.999%) was used as the carrier gas; the carrier gas flow program was set as follows: the initial flow rate was kept at 2.0 mL/min for 2 min, linearly increased to 10.0 mL/min within 8 min, then linearly increased to 100.0 mL/min within 10 min and maintained for another 10 min, with a total GC running time of 30 min and an injector temperature of 80 °C. For ion mobility spectrometry conditions, a tritium source (H) was adopted as the ionization source, the drift tube length was 53 mm with an electric field strength of 500 V/cm and a drift tube temperature of 45 °C, high-purity nitrogen (purity ≥99.999%) served as drift gas at a flow rate of 75.0 mL/min, positive ion mode was selected for detection, the gate opening time was 100 μs, and the gate closing voltage was 90 dgt. For data processing, a series of n-alkane mixed standard solutions were tested to establish calibration curves for retention time and retention index (RI). The retention index of each analyte was subsequently calculated based on its retention time. Qualitative analysis of target compounds was performed by searching and matching against the built-in GC retention index database (NIST 2020) and IMS drift time database in the VOCal software. Plug-ins including Reporter, Gallery Plot and Dynamic PCA in VOCal were applied to generate 3D spectra, 2D spectra, differential spectra, fingerprint spectra and PCA plots of volatile components, so as to compare volatile organic compounds among different samples.

The relative odor activity value (ROAV) of each aroma compound was calculated with Eq. (3) on the basis of relative quantification, as follows:

(3) $$\begin{array}{c} \text{ROAV}=100\times \frac{C_{i}}{T_{i}}\times \frac{T_{\max}}{C_{\max}} \end{array}$$

where Ci, the relative content of aromatic compounds in peanuts (%); T_i_, the aroma threshold of compounds (μg/kg), C_max_ and T_max_, the relative contents of compounds with the greatest contribution to the overall flavor of the sample.

### 2.8 Statistical analysis

Origin (version 9.0) and SPSS (version 21.0) were used for data processing and statistical analysis. The nutritional and flavor measurements were conducted with 3 replicates, and mean values were used for subsequent analysis. One-way analysis of variance (ANOVA) combined with Duncan's multiple range test was applied to compare the differences among groups, and differences were considered significant at *p* < 0.05. In addition, PCA was performed on quality indices of dried peanut pods to reduce data dimensionality and carry out comprehensive evaluation and ranking of different drying treatments.

## 3 Results and discussion

### 3.1 Drying characteristics

Fig. 1 illustrates the drying rates of peanut pods under various drying methods and parameters. During the drying process of HAD, initial moisture loss was primarily attributable to the peanut shells and the surface layer of the peanut kernels. In the later stages of HAD, the primary source of moisture loss shifted to the peanut kernels; consequently, moisture distribution within peanut pods exhibited a direction opposite to that of heat transfer, which was determined by the difference between the moisture diffusion gradient and the temperature gradient. The drying rate was primarily governed by the internal moisture distribution within the peanut pods, leading to a rapid decline in the drying rate (Xie et al., 2023). In contrast to the consistent drying curve of HAD, HASD modified the drying method at the transition point, thus transferring the regulation of the drying rate to control via external conditions. The drying rate increased at this point, marking the point of transition in the drying mechanism. Following the transition in drying method, in IRD process, the electromagnetic wave frequency may have resonated with the molecular frequency of peanut pods, thereby potentially triggering a resonance effect that converted energy into heat. This facilitated rapid and consistent heating and drying of the peanut pods (Zhang et al., 2023). In RFD process, high-frequency electromagnetic fields induced rapid vibrations in the water molecules within peanut pods, thereby generating heat through dielectric heating and directly heating the interior of peanut pods (Wu et al., 2022). HPD employed convective heat transfer, despite the direction of moisture movement and heat transfer remaining in the same direction, the increase in temperature could accelerate the drying rate (Aktaş et al., 2019; Tunckal & Doymaz, 2020). In summary, the adoption of HASD significantly enhanced the drying rates.

**Fig. 1:**
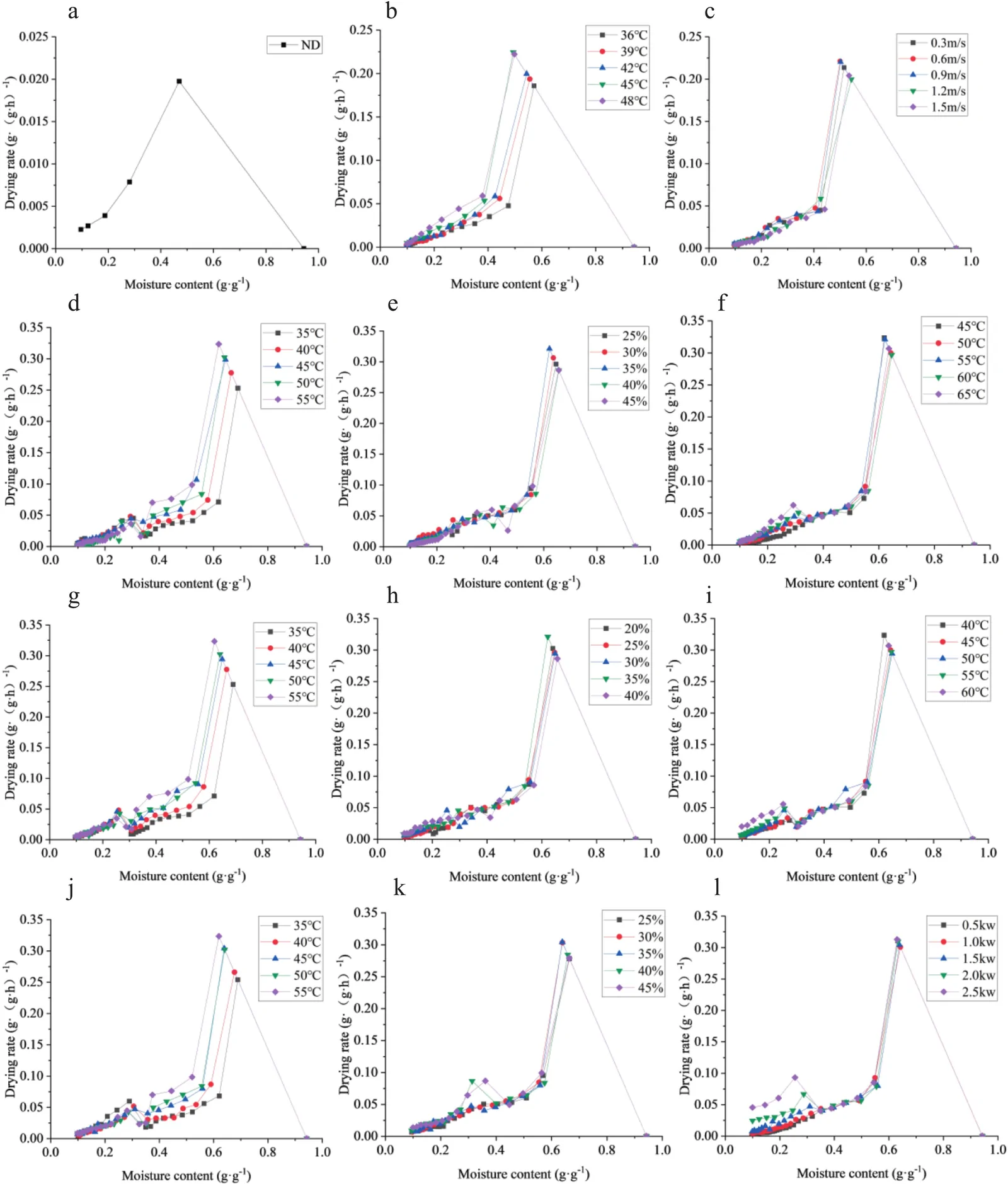
Drying rate of different drying methods of different parameters for peanut pods. (a) ND; (b) HAD: different hot air temperature; (c) HAD: different hot air velocity; (d) HAD-HPD: different hot air temperature; (e) HAD-HPD: different conversion moisture content; (F) HAD-HPD: different heat pump temperature; (g) HAD-IRD: different hot air temperature; (h) HAD-IRD: different conversion moisture content; (i) HAD-IRD: different infrared temperature; (j) HAD-RFD: different hot air temperature; (k) HAD-RFD: different conversion moisture content; (l) HAD-RFD: different RF power.

### 3.2 Microstructure

As demonstrated in Fig. 2, the porous network structure of fresh peanut kernels was characterized by plumpness and regularity, with large pore sizes. Visual comparison of SEM images suggested that the degree of microstructural preservation in dried peanut kernels appeared to follow the order: ND > HAD-HPD > HAD > HAD-IRD > HAD-RFD. In the latter stage of HAD-HPD processing, the HPD provided a gentle and stable drying process due to the near-equivalence between surface moisture evaporation rates and internal moisture migration rates. This process effectively minimized cell wall damage, thereby preserving the microstructural integrity of peanut kernels (Xie et al., 2024). The latter stages of both HAD-IRD and HAD-RFD drying depended on electromagnetic wave-generated heat, which accelerated the contraction of peanut kernel pores and the plastic deformation of cell walls. Among these, radio frequency waves exhibited superior penetrating power, resulting in significant pore crimping and densification, thereby disrupting capillary structures (Li et al., 2024).

**Fig. 2:**
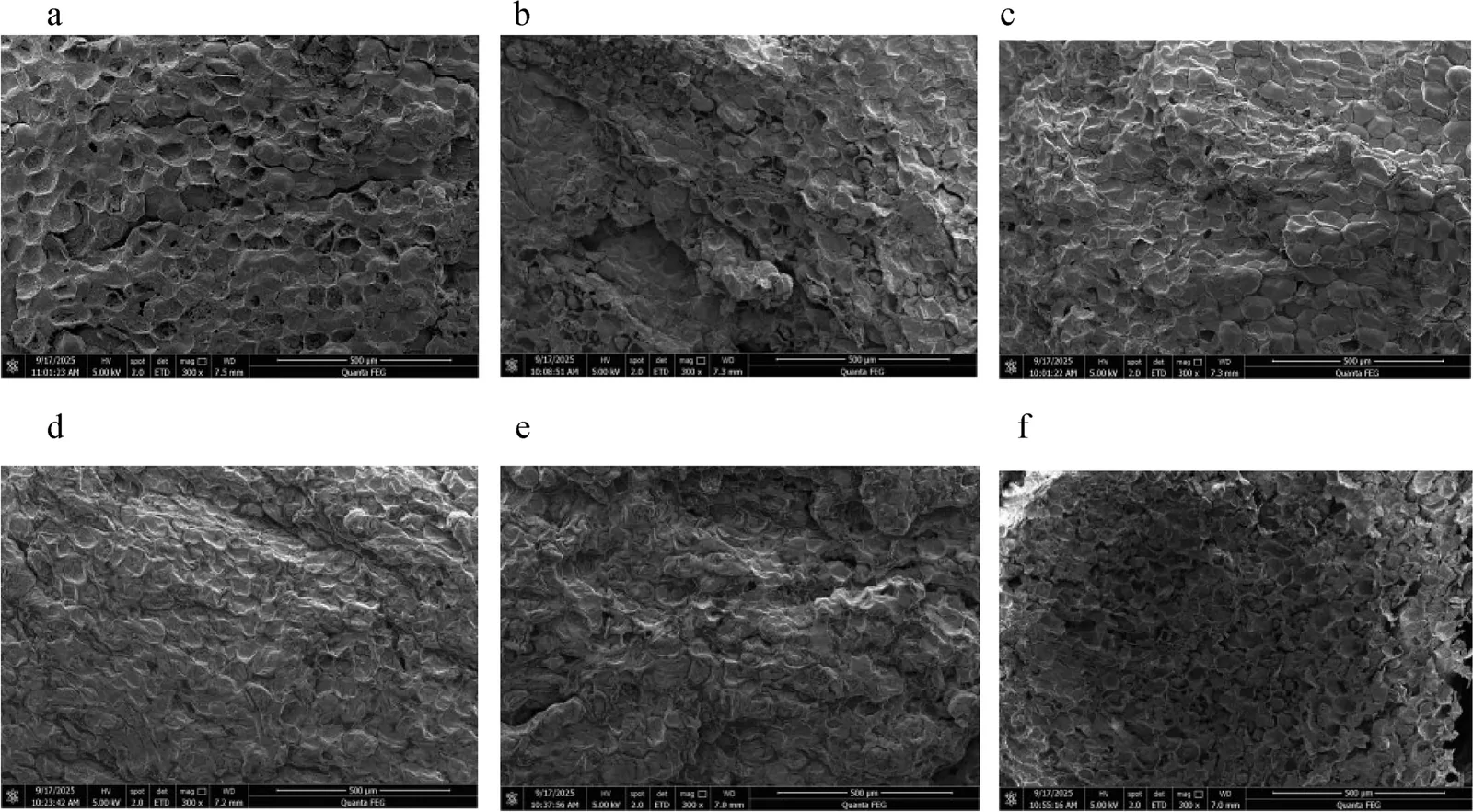
Microstructure of different drying methods for peanut kernels. Fresh; (b) ND; (c) HAD; (d) HAD-HPD; (e) HAD-IRD; (f) HAD-RFD.

### 3.3 Moisture migration

As demonstrated in Fig. 3(a), fresh peanut kernels exhibited a high moisture content that was distributed uniformly throughout the kernel. During HAD, convective heat transfer preferentially removed surface moisture evaporation, with internal moisture migration lagging behind; this created a gradient moisture distribution within the peanut kernel, characterized by a dry outer layer and moist inner layer. For HASD, the HAD-HPD phase exhibited characteristics similar to the preceding HAD phase, with peanut kernels exhibiting an internal moisture gradient between the inner and outer layers. HAD-IRD and HAD-RFD relied on radiant heat transfer, thus enabling synchronous moisture reduction in both the inner and outer layers of peanut kernels. Consequently, at the drying endpoint, the moisture content at the kernel center was lower than that in HAD-HPD. As demonstrated in Fig. 3(b), in comparison to fresh peanut kernels, the relaxation peaks shifted to the left in dried peanut kernels, indicating a reduction in free water content. The high-intensity radio frequency waves in the latter stage of HAD-RFD severely disrupted the microstructure of peanut kernels, weakening the binding forces between bound water and macromolecules. This rendered the water more prone to loss, resulting in the lowest proportion of free water in HAD-RFD-dried peanuts.

**Fig. 3:**
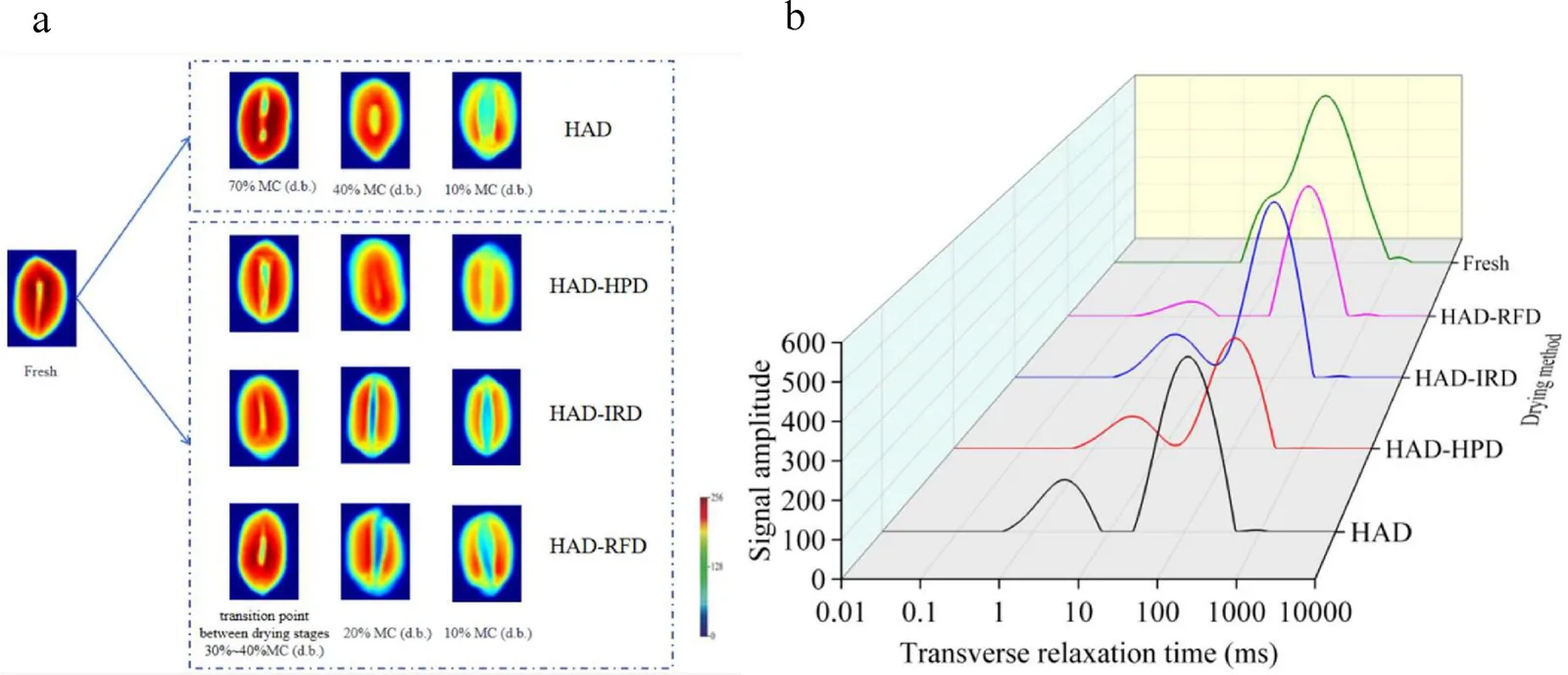
MRI (a) and NMR transverse relaxation time (b) of different drying methods for peanut kernels.

### 3.4 Nutritional quality

As demonstrated in Fig. 4(a), in comparison with HAD, HASD enhanced the retention of crude fat content in dried peanut kernels. Prolonged exposure to elevated temperatures has been shown to enhance the activity of lipoxygenase, thereby accelerating the oxidative breakdown of fats. Consequently, HAD peanut kernels exhibited the lowest crude fat content. Although HAD-RFD significantly reduced the drying time, the subsequent RFD stage tended to cause localized overheating of peanut kernels, accelerating fat oxidation (Wang et al., 2021). Consequently, the crude fat content of HAD-RFD was only marginally higher than that of HAD. IRD demonstrated a capacity to inactivate polyphenol oxidase and lipoxygenase through rapid heating. This process effectively inhibited enzyme-catalyzed oxidation reactions, thereby preserving the antioxidant substances present peanut pods (Dehghan-Manshadi et al., 2020). This resulted in a significantly higher crude fat content in HAD-IRD compared to HAD. The unsaturated fatty acids present in peanut oil were more prone to oxidative deterioration during the drying process, a phenomenon attributable to the presence of carbon‑carbon double bonds, which are less stable (Zhu, Li, et al., 2021). As demonstrated in Table 3, in comparison with HAD, HAD-IRD yielded a higher relative proportion of unsaturated fatty acids in peanut oil. IRD effectively preserved antioxidant-active components within peanut pods (Wu et al., 2022; Zhu, Li, et al., 2021).The mitigation of oxidative damage to carbon‑carbon double bonds in HAD-IRD peanut kernels was achieved through mechanisms such as quenching reactive oxygen species, thereby enhancing the unsaturated fatty acid content. In HAD-IRD peanut kernels, oxidative damage to carbon‑carbon double bonds may have been mitigated through mechanisms such as quenching reactive oxygen species, thereby enhancing the unsaturated fatty acid content. The drying process potentially degraded natural antioxidant components (e.g., vitamin E and polyphenols) in oilseeds, which in turn accelerated lipid oxidation and deterioration. As demonstrated in Fig. 4(b) and Fig. 4(c), HASD notably reduced the acid value and peroxide value of peanut oil, particularly in the case of HAD-IRD, which enhanced the oil's oxidative stability (Tuncel et al., 2017).

**Fig. 4:**
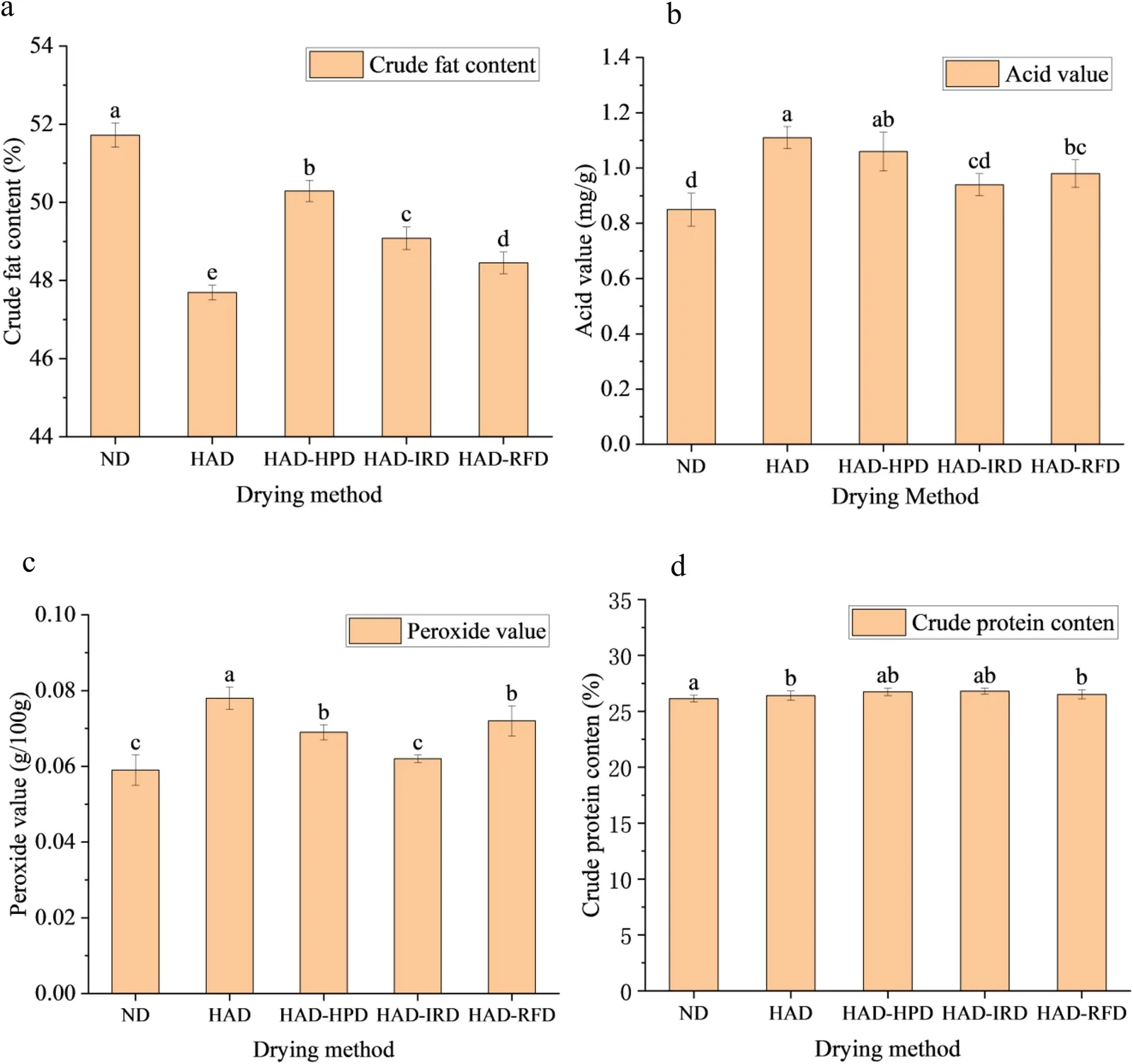
Crude fat content (a), Acid value (b) and peroxide value (c) and Crude protein content (d) of different drying methods for peanut kernels.

**Table 3:** Fatty acid composition and content of different drying methods for peanut kernels.

Fatty acid (g/100 g total fatty acids)	ND	HAD	HAD-HPD	HAD-IRD	HAD-RFD
Palmitic acid (hexadecanoic acid)	11.30 ± 0.09^b^	11.65 ± 0.26^a^	11.28 ± 0.15^b^	11.30 ± 0.13^b^	10.60 ± 0.11^c^
Stearic acid (octadecanoic acid)	4.91 ± 0.09^ab^	4.72 ± 0.31^b^	4.84 ± 0.21^ab^	4.59 ± 0.17^b^	5.11 ± 0.08^a^
Oleic acid (cis-9-octadecenoic acid)	44.65 ± 0.37^b^	43.24 ± 0.36^c^	44.28 ± 0.06^b^	43.69 ± 0.44^c^	48.35 ± 0.07^a^
Linoleic acid (cis-9,12-octadecadienoic acid)	33.68 ± 0.38^c^	34.98 ± 0.23^a^	34.24 ± 0.2^b^	35.19 ± 0.45^a^	30.62 ± 0.13^d^
Linolenic acid (cis-9,12,15-octadecatrienoic acid)	1.42 ± 0.05^c^	1.54 ± 0.01^b^	1.61 ± 0.03^a^	1.36 ± 0.02^d^	1.43 ± 0.03^c^
Arachidic acid (eicosanoic acid)	0.65 ± 0.01^a^	0.58 ± 0.02^bc^	0.46 ± 0.01^d^	0.56 ± 0.03^c^	0.61 ± 0.02^b^
Behenic acid (docosanoic acid)	2.31 ± 0.03^a^	2.14 ± 0.14^b^	2.29 ± 0.03^ab^	2.20 ± 0.08^ab^	2.15 ± 0.05^b^
Tetradecanoic acid (tetradecanoic acid)	1.09 ± 0.02^a^	1.14 ± 0.01^a^	0.99 ± 0.03^b^	1.11 ± 0.05^a^	1.02 ± 0.03^b^
SFA	20.25 ± 0.21^a^	20.24 ± 0.3^a^	19.87 ± 0.15^b^	19.76 ± 0.10^bc^	19.49 ± 0.08^c^
UFA	79.75 ± 0.21^c^	79.76 ± 0.3^c^	80.13 ± 0.15^b^	80.24 ± 0.10^ab^	80.51 ± 0.08^a^

Different lowercase letters indicate significant differences (*p* < 0.05).

As demonstrated in Fig. 4(d), in comparison with ND, the crude protein content of dried peanut kernels increased by 1.03% and 1.45% in the HAD and HAD-RFD groups, respectively. No significant difference was observed between the HAD-HPD and HAD-IRD treatment groups (*p* > 0.05). This variation may be attributed to the divergent effects of drying methodologies on protease activity in peanut kernels. Relevant studies have indicated that HPD and IRD are more conducive to preserving the crude protein content of dried materials (Bai et al., 2018; Hassan et al., 2016). As shown in Table 4, compared with ND, both HAD and HASD peanut kernels exhibited reduced amino acid content, indicating that HAD negatively affects amino acid retention (Zhang, Zhao, et al., 2025). In comparison with HAD, HAD-IRD effectively reduced the drying time; concurrently, compared with HAD-RFD, it enhanced drying uniformity, thereby preventing excessive Maillard reactions caused by localized overheating, thus achieving the highest amino acid content.

**Table 4:** Amino acid composition and content of different drying methods for peanut kernels.

Amino acid content(%)	ND	HAD	HAD-HPD	HAD-IRD	HAD-RFD
Asp	3.75 ± 0.1^a^	2.61 ± 0.03^d^	2.77 ± 0.06^c^	2.95 ± 0.03^b^	2.83 ± 0.07^bc^
Thr^⁎^	0.55 ± 0.02^a^	0.46 ± 0.04^b^	0.45 ± 0.04^b^	0.47 ± 0.01^b^	0.49 ± 0.02^b^
Ser	1.22 ± 0.05^a^	0.92 ± 0.03^b^	0.95 ± 0.04^b^	1.00 ± 0.02^b^	1.00 ± 0.04^b^
Glu	7.92 ± 0.26^a^	5.48 ± 0.16^c^	5.88 ± 0.25b^c^	6.25 ± 0.09^b^	5.92 ± 0.21^bc^
Gly	2.08 ± 0.13^a^	1.50 ± 0.04^c^	1.63 ± 0.06^bc^	1.74 ± 0.09^b^	1.67 ± 0.05^bc^
Ala	1.64 ± 0.14^a^	1.10 ± 0.07^c^	1.20 ± 0.03^bc^	1.28 ± 0.02^b^	1.22 ± 0.04^bc^
Cys	0.12 ± 0.02	0.15 ± 0.02	0.15 ± 0.02	0.15 ± 0.02	0.11 ± 0.03
Val*	1.16 ± 0.04^a^	0.80 ± 0.03^c^	0.89 ± 0.05^b^	0.93 ± 0.03^b^	0.87 ± 0.04^bc^
Met*	0.31 ± 0.03^a^	0.26 ± 0.01^b^	0.28 ± 0.01^b^	0.31 ± 0.01^a^	0.25 ± 0.02^b^
Ile*	1.15 ± 0.05^a^	0.78 ± 0.04^c^	0.84 ± 0.04^bc^	0.90 ± 0.03^b^	0.86 ± 0.07^bc^
Leu*	2.12 ± 0.09^a^	1.47 ± 0.03^c^	1.59 ± 0.1^bc^	1.64 ± 0.05^b^	1.62 ± 0.02^bc^
Tyr	1.38 ± 0.08^a^	0.93 ± 0.07^c^	1.01 ± 0.04^bc^	1.08 ± 0.05^b^	1.04 ± 0.1^bc^
Phe*	1.65 ± 0.04^a^	1.09 ± 0.02^c^	1.18 ± 0.04^bc^	1.26 ± 0.06^b^	1.20 ± 0.03^b^
His*	0.95 ± 0.04^a^	0.70 ± 0.08^b^	0.75 ± 0.04^b^	0.80 ± 0.04^b^	0.78 ± 0.06^b^
Lys*	1.21 ± 0.08^a^	0.86 ± 0.05^b^	0.90 ± 0.03^b^	0.97 ± 0.05^b^	0.93 ± 0.07^b^
Arg	4.51 ± 0.18^a^	3.01 ± 0.11^c^	3.14 ± 0.16^c^	3.50 ± 0.08^b^	3.01 ± 0.19^c^
Pro	1.37 ± 0.1^a^	1.02 ± 0.09^b^	1.08 ± 0.06^b^	1.14 ± 0.05^b^	1.12 ± 0.08^b^
Essential amino-acid	9.11 ± 0.05^a^	6.40 ± 0.11^d^	6.87 ± 0.08^c^	7.28 ± 0.05^b^	7.00 ± 0.17^c^
Non-essential amino acid	23.98 ± 0.45^a^	16.71 ± 0.34^d^	17.81 ± 0.5^c^	19.09 ± 0.23^b^	17.92 ± 0.3^c^
Total amino acid	33.09 ± 0.48^a^	23.11 ± 0.44^d^	24.68 ± 0.43^c^	26.37 ± 0.2^b^	24.91 ± 0.45^c^

Different lowercase letters indicate significant differences (*p* < 0.05).

### 3.5 Flavor quality

Fig. 5(a) and 5(b) show the GC-IMS spectra of volatile organic compounds (VOCs) in peanut kernels dried using different methods. Identical retention time and drift time coordinates correspond to the same VOC, with the color indicating its concentration level. While the types of VOCs in peanut kernels were similar across drying methods, their relative content exhibited significant variation (Zhang et al., 2022). All GC-IMS signal intensities were normalized based on sample mass before quantitative comparison. Among these, the signal peak intensity of HASD peanut kernels was significantly higher than that of ND and HAD, with HAD-RFD exhibiting the most pronounced difference. HASD employed two distinct drying techniques to facilitate thorough internal reactions within peanut kernels, thereby generating a greater abundance of flavor compounds. Fig. 5(c) shows that the total peak heights of VOCs in peanut kernels from different drying methods are, in descending order: HAD-RFD, HAD-HPD, HAD, ND, and HAD-IRD. Alcohols exhibited the highest content among the various VOCs, accounting for around 34%, followed by aldehydes at approximately 24% and ketones at 21%. A total of 57 VOCs were detected in dried peanut kernels based on analysis of migration time and retention time features combined with database comparison (substance suffixes M and D denote monomers and dimers of the same substance, which are counted as a single substance). Of these, 9 substances remained unidentified (denoted by numbers) and 48 were identified. These substances comprised 17 aldehydes, 13 alcohols, 8 ketones, 5 esters, 4 heterocyclic compounds, and 1 acid. Aldehydes were the most abundant VOCs in peanut kernels (Chen, Wang, Wang, et al., 2025). Fig. 5(e) provides a visual comparison of the differences between the VOC fingerprint spectra of peanut kernels processed by different drying methods. Each bright spot in the figure represents a distinct VOC, with redder hues indicating higher concentrations. The red frames labeled A to E in Fig. 5(e) correspond to the characteristic VOCs found in peanut kernels dried using different methods. ND exhibited the greatest variety of characteristic VOCs; as the adoption of other drying methods, the number of characteristic VOCs decreased. A total of 16 characteristic VOCs were identified in ND, dominated by aldehydes. HAD-RFD had 14 characteristic VOCs with a relatively diverse composition, including ketones, aldehydes, and heterocyclic compounds. HAD-HPD contained 11 characteristic VOCs, which were mainly alcohols. Two characteristic VOCs were detected in both HAD and HAD-IRD.

**Fig. 5:**
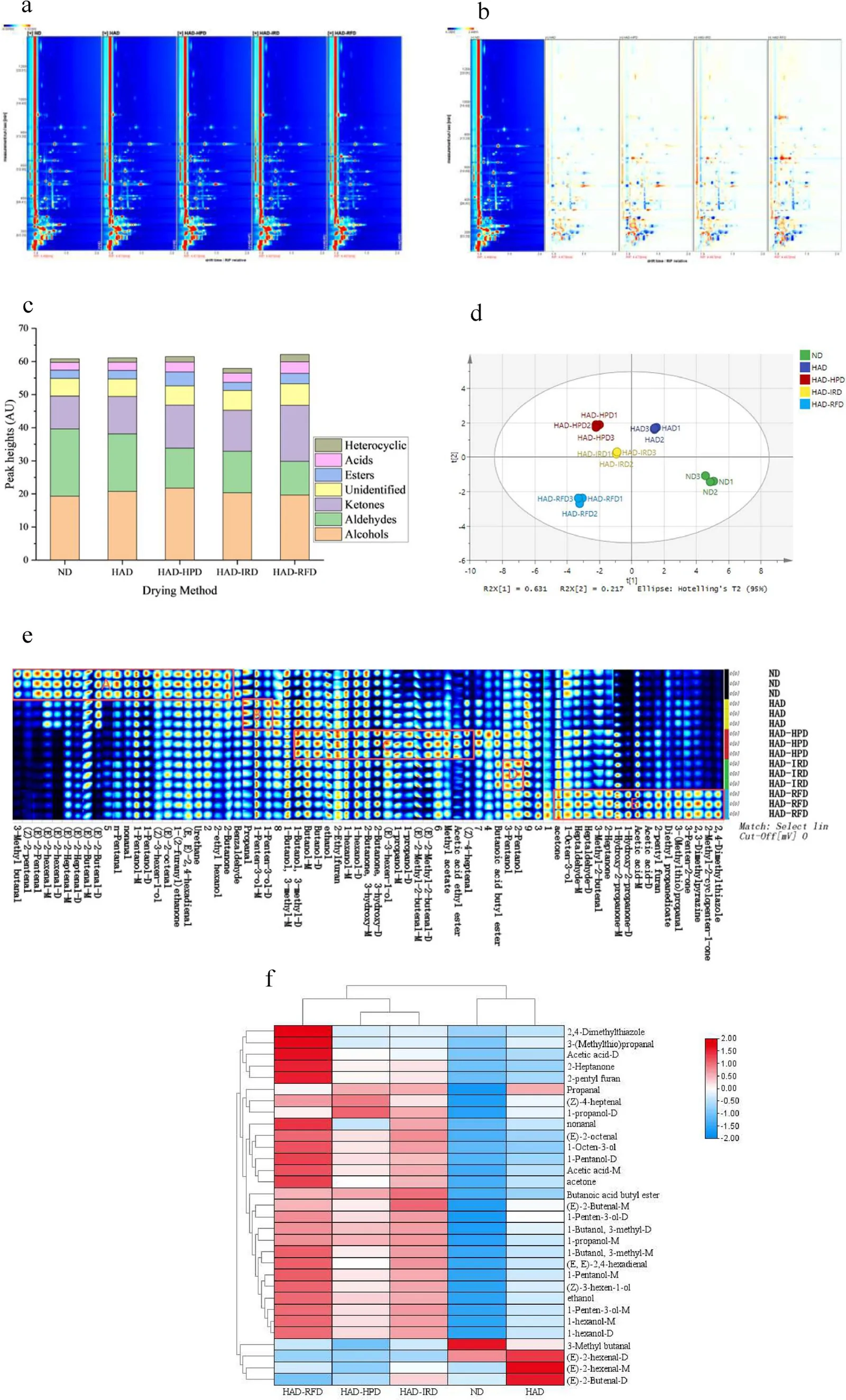
Analysis and characterization of VOCs in peanut kernels via different drying methods. (a) GC-IMS 2D map; (b) GC-IMS difference map; (c) VOC peak height distribution; (d) GC-IMS fingerprint spectra of VOCs; (e) PLS-DA of VOCs; (f) Heatmap of key VOCs.

Aldehydes are the most important VOCs due to their relatively high content and low odor threshold. As shown in Fig. 5(c), after ND, HAD, HAD-HPD, HAD-IRD, and HAD-RFD, the proportions of aldehydes in peanut kernel VOCs were 33%, 28%, 20%, 22%, and 16%, respectively. HASD resulted in a significant decrease in aldehyde content in peanut kernels, particularly for HAD-RFD. After HASD, aldehydes in peanut kernels were mainly converted into ketones: the high temperature of hot-air drying promotes lipid-derived aldehydes to undergo aldol condensation, dehydration and carbonyl rearrangement reactions to realize the conversion from aldehydes to ketones; in addition, aldehydes in HAD-HPD and HAD-IRD were partially converted into alcohols. Both ketones and alcohols are mainly derived from the oxidation of fats during heating. Among all drying methods, HAD-RFD involved the most intense drying conditions, leading to the highest degree of aldehyde conversion. In contrast, IRD in the later stage of HAD-IRD exhibited antioxidant effects, resulting in better retention of aldehydes in this group (Bouhile et al., 2025). Among the VOCs in dried peanut kernels, (E)-2-Butenal was one of the most abundant VOCs for ND and HAD. HASD caused a significant reduction in (E)-2-Butenal content, with the reduction rates reaching 75% (HAD-HPD), 63% (HAD-IRD), and 88% (HAD-RFD), respectively. During drying, the double bonds and aldehyde groups of (E)-2-Butenal were prone to oxidation to form compounds such as ketones, which were further converted into small-molecule VOCs including Acetone, Acetic acid, 1-Propanol, Methyl acetate, 1-Hydroxy-2-propanone, and Propanal. Among these compounds, Acetone was the most abundant VOC in dried peanut kernels. Compared with ND and HAD, the Acetone content was higher in all HASD treatments, especially for HAD-RFD, where it increased by 78% relative to ND. Although both RFD and IRD generate heat via electromagnetic radiation, radio frequency waves with longer wavelengths penetrate deeper into peanut kernels, enabling more thorough reactions. In addition, RFD tended to cause edge scorching of peanut kernels (Huang et al., 2018), which intensified the Maillard reaction and promoted the formation of large amounts of substances such as Acetic acid and 2,4-Dimethylthiazole, endowing the peanut kernels with pungent, roasted, and grilled-meat flavors. In summary, the peanut kernels treated with HAD-RFD drying exhibited the highest diversity of VOCs.

As shown in Fig. 5(d), PLS-DA analysis of the VOC profiles from dried peanut kernels revealed that PC1 and PC2 accounted for 63.1% and 21.7% of the total variance, respectively, with a combined contribution rate of 84.8%. This suggested that these 2 principal components encompassed most of the flavor information present in dried peanut kernels. The results indicated that GC-IMS technology can distinguish between peanut kernel samples dried by different methods.

The flavor profile of peanut kernels was determined by the content and aroma threshold of their constituent VOCs. It was evident that the greater the ROAV value, the more significant the contribution of the corresponding VOC to the overall flavor profile of peanut kernels. It is widely accepted that VOCs exhibiting a ROAV greater than 1 are key VOCs in the material (Zhu, Niu, & Xiao, 2021). After drying, 3-methyl butanal was identified as the predominant key VOC in ND peanut kernels. In HAD, HAD-HPD, and HAD-IRD peanut kernels, propanal was identified as the predominant key VOC. In HAD-RFD peanut kernels, the predominant key VOC was identified as 3-methyl-1-butanol. As demonstrated in Table 5, a total of 23 key VOCs were identified in dried peanut kernels (substance suffixes M and D denote monomers and dimers of the same substance, which are counted as a single substance): ND (12 compounds), HAD (20 compounds), HAD-HPD (22 compounds), HAD-IRD (22 compounds), and HAD-RFD (22 compounds). As represented in Fig. 5(f), the key VOC data were categorized into two primary clusters following cluster analysis, with all HASD-treated samples clustered in the same group. Within the HASD framework, the HAD-RFD could be further categorized as a distinct subset. In the context of the 5 drying methods, the following 3 VOCs were identified as the most prevalent in dried peanut kernels: propanal, which possesses a pungent and grassy aroma; 1-butanol, exhibiting both whisky-like and banana-like fruit notes; 3-methyl, and (E)-2-hexenal, which exhibits a grassy, banana-like flavor with fatty undertones. Collectively, these compounds were responsible for the most significant flavor characteristics of dried peanut kernels. Furthermore, the peanut kernels of ND were characterized by a chocolate aroma; those of HAD exhibited a fatty aroma; HAD-HPD peanut kernels displayed notes of alcohol, freshness, and grassiness; HAD-IRD kernels carried floral and fruity scents, while HAD-RFD kernels featured pungency, roasted aromas, and a smoky, grilled character.

**Table 5:** ROAV of Key VOCs of different drying methods for peanut kernels.

Count	Compound	Threshold in air mg/m^3^	ROAV					Odor Description
			ND	HAD	HAD-HPD	HAD-IRD	HAD-RFD	
1	Propanal	0.0048	38.04	100.00	100.00	100.00	87.01	pungent, green grassy
2	1-Butanol, 3-methyl-D	0.0061	18.45	67.56	88.71	96.85	100.00	whiskey, banana, fruity
3	1-Butanol, 3-methyl-M	0.0061	21.69	48.44	63.15	77.41	88.16	whiskey, banana, fruity
4	3-Methyl butanal	0.0010	100.00	58.58	24.54	43.17	42.06	chocolate, fat
5	Acetic acid-M	0.0130	14.40	37.86	56.07	66.70	83.73	spicy
6	(E)-2-hexenal-M	0.0031	42.48	81.42	37.11	50.37	45.55	green, banana, fat
7	2-Heptanone	0.0035	5.15	8.81	19.31	20.50	37.08	pear, banana, fruity, slight medicinal fragrance
8	(E)-2-octenal	0.0027	7.63	12.24	19.46	23.91	25.89	fresh cucumber, fatty, green herbal, banana, green leaf
9	(E)-2-hexenal-D	0.0031	31.23	41.03	5.00	6.58	4.99	green, banana, fat
10	nonanal	0.0031	7.11	10.26	10.27	14.73	18.01	rose, citrus, strong oily
11	(E, E)-2,4-hexadienal	0.0018	4.49	9.90	11.90	15.65	17.46	sweet, green, floral, citrus
12	1-hexanol-M	0.0340	4.24	10.02	12.92	15.03	16.92	fresh, fruity, wine, sweet, green
13	1-Penten-3-ol-M	0.0500	3.12	7.69	10.19	12.15	13.39	ethereal, green, tropical fruity
14	1-hexanol-D	0.0340	3.00	7.26	9.73	11.21	12.61	fresh, fruity, wine, sweet, green
15	Acetic acid-D	0.0130	1.11	3.55	7.72	7.13	19.56	spicy
16	(E)-2-Butenal-M	0.0670	3.23	7.49	7.96	10.45	8.99	pungent
17	(E)-2-Butenal-D	0.0670	6.19	14.33	4.98	8.80	3.24	pungent
18	2-pentyl furan	0.0190	0.62	2.70	5.93	6.44	13.00	bean, fruity, earthy, green, vegetable
19	1-Penten-3-ol-D	0.0500	0.86	3.58	3.96	4.68	4.84	ethereal, green, tropical fruity
20	2,4-Dimethylthiazole	0.0180	0.26	1.19	1.76	1.93	8.67	baking, roasted meat
21	1-Pentanol-M	0.1530	0.96	2.13	2.69	3.11	3.67	balsamic
22	Butanoic acid butyl ester	0.0280	0.45	1.32	3.25	3.83	3.13	fruity, banana, pineapple, green cherry, tropical fruit mature and juicy fruity aroma
23	1-propanol-M	0.2400	0.57	1.89	2.87	3.09	3.14	alcohol, pungent
24	1-Octen-3-ol	0.0110	0.92	1.30	2.41	2.95	3.53	mushroom, lavender, rose, hay
25	1-Pentanol-D	0.1530	0.84	1.27	2.01	2.19	2.86	balsamic
26	(Z)-4-heptenal	0.0310	0.26	1.41	2.17	1.64	2.02	grass, oil
27	acetone	0.8320	0.30	0.96	1.33	1.70	2.31	fragrant
28	ethanol	0.6200	0.44	1.09	1.45	1.59	1.84	aromaticity
29	(Z)-3-hexen-1-ol	0.0130	0.50	1.01	1.18	1.37	1.55	green, herb
30	1-propanol-D	0.2400	0.12	0.76	1.32	1.10	0.89	alcohol, pungent
31	3-(Methylthio)propanal	0.0600	0.13	0.56	0.52	0.56	1.71	onion, meat, fruity

### 3.6 Comprehensive evaluation

The quality data of dried peanut pods were subjected to principal component analysis (PCA) for dimensionality reduction using SPSS. As shown in Table 6, PC1 accounted for 54.57% of the variation and was primarily correlated with acid value, peroxide value, crude protein content, and amino acid content. PC2 contributed 30.71%, primarily associated with crude fat content, unsaturated fatty acid proportion, and total VOC peak height. The variance contribution of PC1 was higher than that of PC2, indicating its more pronounced influence on the quality under the four drying methods. PC1 and PC2 together accounted for more than 80% of the total variance, indicating that the selected principal components effectively characterized the comprehensive quality of peanut pods dried by the four methods. A comprehensive evaluation ranking of the four drying methods was established based on the cumulative contribution rates of the two principal components presented in Table 6. The comprehensive scores and ranking results indicate that the overall quality ranking of the four drying methods is as follows: HAD-IRD > HAD-HPD > HAD-RFD > HAD. HAD-IRD yielded the highest comprehensive score.

**Table 6:** Principal component scores and comprehensive evaluation results.

Drying method	Principal Component Scores		Comprehensive Score	Ranking
	F1	F2		
HAD-IRD	1.215	0.823	90.5	1
HAD-HPD	0.987	1.012	84.2	2
HAD-RFD	0.764	0.958	74.6	3
HAD	−0.892	−0.745	13.8	4

## 4 Conclusion

The present study systematically compared the effects of ND, HAD, and HASD (HAD-HPD, HAD-IRD, HAD-RFD) on the drying characteristics, microstructure, moisture migration, nutritional quality, and flavor quality of peanut pods. The results of the study indicated that the HASD process significantly enhanced peanut pod drying rates, improved their nutritional quality, and enhanced their flavor profiles. For drying characteristics, HASD exhibited a drying mechanism transition point, where drying rates underwent significant changes during the switch between convective and radiative drying modes. In terms of microstructure, HAD-HPD exhibited optimal integrity, while HAD-IRD and HAD-RFD exhibited severe deformation of cell walls. With regard to moisture migration, both HAD-IRD and HAD-RFD were found to reduce both internal and external peanut kernel moisture during the latter drying stages. Owing to extensive structural damage, peanut kernels dried by HAD-RFD had the lowest free water content at the drying endpoint. In terms of nutritional quality, HAD-IRD exhibited the highest amino acid content, alongside the lowest acid value and peroxide value. The highest crude fat content was exhibited by HAD-HPD. With regard to flavor compounds, HAD-RFD promoted the formation of volatile flavor compounds, yielding distinctive roasted and baked notes, but it also generated undesirable odors. In contrast, HAD-IRD maintained a favorable balance of fresh and fruity aromas. Principal component analysis suggested that HASD was superior to HAD, and HAD-IRD performed best among them. Nevertheless, this study has some limitations. It only investigated the quality and structural properties of dried peanut pods without economic benefit analysis. Future work will evaluate the economic feasibility and industrial application potential of the HAD-IRD process to facilitate its industrial promotion.

## CRediT authorship contribution statement

**Pengxiao Chen:** Formal analysis, Data curation, Conceptualization. **Siyuan Zhao:** Writing – review & editing, Writing – original draft, Project administration. **Wenxue Zhu:** Investigation, Funding acquisition. **Tingting Zhang:** Resources, Project administration, Methodology. **Kai Zhang:** Validation, Supervision. **Yanjia Xing:** Project administration, Methodology. **Can Hua:** Resources, Project administration. **Debang Zhang:** Validation, Investigation.

## Fundings

This work was financially supported by Open Project of Provincial Key Laboratory for Grain Resource Transformation and Utilization (No. 24400017), Open Project of the Key Laboratory for Primary Processing of Agricultural Products in the Production Areas of the Ministry of Agriculture and Rural Affairs (No. KLAPPP-2023-02) and Research and Development of Drying Technology and Equipment for Bulk Oilseed Crops (No. 2024TXTD09).

## Declaration of competing interest

The authors declare that they have no known competing financial interests or personal relationships that could have appeared to influence the work reported in this paper.

## Data Availability

I have shared the data in the attachement.
