# Supplementary material for: Comparison of different hot air segmented drying techniques for peanut pods: changes in drying characteristics, microstructure, moisture migration, nutritional quality and flavor quality
Source: Food Chem X. 2026 Sep 29;39:104533. doi: 10.1016/j.fochx.2026.104533 (PMC13683451; doi:10.1016/j.fochx.2026.104533)
Supplement: Supplementary file 1 [file mmc1.docx]

**Supplementary Material**

a	b	c

d	e	f

g	h	i

j	k	l

Fig.7. Moisture content of different drying methods of different parameters for peanut pods.(a) ND; (b) HAD: different hot air temperature; (c) HAD: different hot air velocity; (d) HAD-HPD: different hot air temperature; (e) HAD-HPD: different conversion moisture content; (F) HAD-HPD: different heat pump temperature; (g) HAD-IRD: different hot air temperature; (h) HAD-IRD: different conversion moisture content; (i) HAD-IRD: different infrared temperature; (j) HAD-RFD: different hot air temperature; (k) HAD-RFD: different conversion moisture content; (l) HAD-RFD: different RF power.
