# Supplementary material for: Comparison of different hot air segmented drying techniques for peanut pods: changes in drying characteristics, microstructure, moisture migration, nutritional quality and flavor quality
Source: Food Chem X. 2026 Sep 29;39:104533. doi: 10.1016/j.fochx.2026.104533 (PMC13683451; doi:10.1016/j.fochx.2026.104533)
Supplement: Supplementary file 2 [file mmc2.docx]

**Supplementary Material**

Table 7 The information on detected VOCs of peanut kernels of different drying methods by GC-IMS.

| Categories | Count | Compound | CAS# | Formula | M_W_ | Rt [sec] | Dt [a.u.] |
| --- | --- | --- | --- | --- | --- | --- | --- |
|  |  |  |  |  |  |  |  |
| Aldehydes | 1 | Benzaldehyde | C100527 | C7H6O | 106.1 | 972.04 | 1.15 |
|  | 2 | (E)-2-octenal | C2548870 | C8H14O | 126.2 | 820.83 | 1.33 |
|  | 3 | (E)-2-Heptenal-D | C18829555 | C7H12O | 112.2 | 678.52 | 1.65 |
|  | 4 | (E)-2-Heptenal-M | C18829555 | C7H12O | 112.2 | 680.41 | 1.26 |
|  | 5 | (E)-2-hexenal-D | C6728263 | C6H10O | 98.1 | 504.99 | 1.51 |
|  | 6 | (E)-2-hexenal-M | C6728263 | C6H10O | 98.1 | 506.43 | 1.18 |
|  | 7 | (Z)-4-heptenal | C6728310 | C7H12O | 112.2 | 546.19 | 1.14 |
|  | 8 | 3-Methyl-2-butenal | C107868 | C5H8O | 84.1 | 476.44 | 1.09 |
|  | 9 | (E)-2-Butenal-M | C123739 | C4H6O | 70.1 | 292.24 | 1.04 |
|  | 10 | (E)-2-Butenal-D | C123739 | C4H6O | 70.1 | 292.49 | 1.2 |
|  | 11 | 3-(Methylthio)propanal | C3268493 | C4H8OS | 104.2 | 867.74 | 1.09 |
|  | 12 | (E, E)-2,4-hexadienal | C142836 | C6H8O | 96.1 | 827.94 | 1.11 |
|  | 13 | nonanal | C124196 | C9H18O | 142.2 | 772.42 | 1.5 |
|  | 14 | Heptaldehyde-M | C111717 | C7H14O | 114.2 | 446.14 | 1.37 |
|  | 15 | Heptaldehyde-D | C111717 | C7H14O | 114.2 | 445.94 | 1.69 |
|  | 16 | (E)-2-Pentenal | C1576870 | C5H8O | 84.1 | 377.61 | 1.36 |
|  | 17 | (Z)-2-pentenal | C1576869 | C5H8O | 84.1 | 363.97 | 1.09 |
|  | 18 | (E)-2-Methyl-2-butenal-M | C497030 | C5H8O | 84.1 | 335.67 | 1.11 |
|  | 19 | (E)-2-Methyl-2-butenal-D | C497030 | C5H8O | 84.1 | 336.67 | 1.35 |
|  | 20 | n-Pentanal | C110623 | C5H10O | 86.1 | 238.8 | 1.42 |
|  | 21 | Propanal | C123386 | C3H6O | 58.1 | 146.52 | 1.14 |
|  | 22 | 3-Methyl butanal | C590863 | C5H10O | 86.1 | 196.7 | 1.41 |
| Alcohol | 23 | 2-Octanol-M | C123966 | C8H18O | 130.2 | 831.23 | 1.43 |
|  | 24 | 2-Octanol-D | C123966 | C8H18O | 130.2 | 829.33 | 1.81 |
|  | 25 | 2-ethyl hexanol | C104767 | C8H18O | 130.2 | 1008.9 | 1.4 |
|  | 26 | (E)-3-hexen-1-ol | C928972 | C6H12O | 100.2 | 771.16 | 1.26 |
|  | 27 | 1-hexanol-D | C111273 | C6H14O | 102.2 | 729.77 | 1.65 |
|  | 28 | 1-hexanol-M | C111273 | C6H14O | 102.2 | 729.77 | 1.33 |
|  | 29 | 1-Pentanol-D | C71410 | C5H12O | 88.1 | 564.25 | 1.52 |
|  | 30 | 1-Pentanol-M | C71410 | C5H12O | 88.1 | 564.25 | 1.26 |
|  | 31 | 1-Butanol, 3-methyl-D | C123513 | C5H12O | 88.1 | 487.28 | 1.5 |
|  | 32 | 1-Butanol, 3-methyl-M | C123513 | C5H12O | 88.1 | 486.56 | 1.25 |
|  | 33 | 1-Penten-3-ol-M | C616251 | C5H10O | 86.1 | 415.95 | 0.94 |
|  | 34 | Butanol-D | C71363 | C4H10O | 74.1 | 392.71 | 1.39 |
|  | 35 | Butanol-M | C71363 | C4H10O | 74.1 | 393.3 | 1.19 |
|  | 36 | ethanol | C64175 | C2H6O | 46.1 | 215.15 | 1.12 |
|  | 37 | 1-Octen-3-ol | C3391864 | C8H16O | 128.2 | 882.11 | 1.16 |
|  | 38 | (Z)-3-hexen-1-ol | C928961 | C6H12O | 100.2 | 789.75 | 1.24 |
|  | 39 | 2-Pentanol | C6032297 | C5H12O | 88.1 | 364.48 | 1.22 |
|  | 40 | 3-Pentanol | C584021 | C5H12O | 88.1 | 350.49 | 1.21 |
|  | 41 | 1-propanol-D | C71238 | C3H8O | 60.1 | 278.34 | 1.26 |
|  | 42 | 1-propanol-M | C71238 | C3H8O | 60.1 | 275.86 | 1.11 |
|  | 43 | 1-Penten-3-ol-D | C616251 | C5H10O | 86.1 | 414.16 | 1.39 |
| Ketones | 44 | acetone | C67641 | C3H6O | 58.1 | 165.6 | 1.11 |
|  | 45 | 1-(2-furanyl) ethanone | C1192627 | C6H6O2 | 110.1 | 1087.34 | 1.12 |
|  | 46 | 1-Hydroxy-2-propanone-M | C116096 | C3H6O2 | 74.1 | 651.51 | 1.11 |
|  | 47 | 2-Butanone, 3-hydroxy-D | C513860 | C4H8O2 | 88.1 | 625.13 | 1.33 |
|  | 48 | 1-Hydroxy-2-propanone-D | C116096 | C3H6O2 | 74.1 | 652.19 | 1.23 |
|  | 49 | 2-Methyl-2-cyclopenten-1-one | C1120736 | C6H8O | 96.1 | 749.63 | 1.12 |
|  | 50 | 2-Butanone, 3-hydroxy-M | C513860 | C4H8O2 | 88.1 | 628.53 | 1.09 |
|  | 51 | 2-Heptanone | C110430 | C7H14O | 114.2 | 441.66 | 1.27 |
|  | 52 | 3-Penten-2-one | C625332 | C5H8O | 84.1 | 375.39 | 1.07 |
|  | 53 | 2-Butanone | C78933 | C4H8O | 72.1 | 196.08 | 1.24 |
| Esters | 54 | Urethane | C51796 | C3H7NO2 | 89.1 | 1317.83 | 1.1 |
|  | 55 | Methyl acetate | C79209 | C3H6O2 | 74.1 | 173.76 | 1.19 |
|  | 56 | Diethyl propanedioate | C105533 | C7H12O4 | 160.2 | 1269.14 | 1.26 |
|  | 57 | Butanoic acid butyl ester | C109217 | C8H16O2 | 144.2 | 530.15 | 1.33 |
|  | 58 | Acetic acid ethyl ester | C141786 | C4H8O2 | 88.1 | 180.79 | 1.33 |
| Heterocyclic | 59 | 2,3-Dimethylpyrazine | C5910894 | C6H8N2 | 108.1 | 703.76 | 1.12 |
|  | 60 | 2-pentyl furan | C3777693 | C9H14O | 138.2 | 517.24 | 1.25 |
|  | 61 | 2,4-Dimethylthiazole | C541582 | C5H7NS | 113.2 | 587.14 | 1.1 |
|  | 62 | 2-Ethylfuran | C3208160 | C6H8O | 96.1 | 235.51 | 1.31 |
| Acid | 63 | Acetic acid-M | C64197 | C2H4O2 | 60.1 | 904.94 | 1.06 |
|  | 64 | Acetic acid-D | C64197 | C2H4O2 | 60.1 | 906.21 | 1.17 |
